# Inhibition of autophagy triggers melatonin-induced apoptosis in glioblastoma cells

**DOI:** 10.1186/s12868-019-0545-1

**Published:** 2019-12-23

**Authors:** Nan Zhou, Zi Xuan Wei, Zeng Xin Qi

**Affiliations:** Department of Neurosurgery, Huashan Hospital, Fudan University, Middle Urumqi Road 12, Shanghai, 200040 China

**Keywords:** Melatonin, Agomelatine, Autophagy, Apoptosis, Glioma

## Abstract

**Background:**

Autophagy is considered to be another restorative focus for the treatment of brain tumors. Although several research have demonstrated that melatonin induces autophagy in colon cancer and hepatoma cells, there has not been any direct evidence of whether melatonin is capable of inducing autophagy in human glioma cells.

**Results:**

In the present research, we report that melatonin or its agonist, agomelatine, induced autophagy in A172 and U87-MG glioblastoma cells for a concentration-and time-dependent way, which was significantly attenuated by treatment with luzindole, a melatonin receptor antagonist. Furthermore, by suppressing autophagy at the late-stage with bafilomycin A1 and early stage with 3-MA, we found that the melatonin-induced autophagy was activated early, and the autophagic flux was complete. Melatonin treatment alone did not induce any apoptotic changes in the glioblastoma cells, as measured by flow cytometry. Western blot studies confirmed that melatonin alone prominently upregulated the levels of Beclin 1 and LC3 II, which was accompanied by an increase in the expression of Bcl-2, whereas it had no effect on the expression of Bax in the glioblastoma cells. Remarkably, co-treatment with 3-MA and melatonin significantly enhanced the apoptotic cell population in the glioblastoma cells, along with a prominent decrease in the expression of bcl-2 and increase in the Bax expression levels, which collectively indicated that the disruption of autophagy triggers the melatonin-induced apoptosis in glioblastoma cells.

**Conclusions:**

These results provide information indicating that melatonin may act as a common upstream signal between autophagy and apoptosis, which may lead to the development of new therapeutic strategies for glioma.

## Background

Malignant glioblastomas (GBMs) are the most common form of brain tumors and are associated with a poor prognosis [1]. The standard treatment for GBMs is surgical resection of the tumor followed by concurrent radiation therapy (RT) and chemotherapy with temozolomide (TMZ). Autophagy, as a highly conserved cellular homeostatic process that can have either a tumor suppressor or promoter effect depending on the tumor type and stage, is considered to be a new target for therapeutic intervention in brain tumors [2]. Interestingly, both TMZ and RT induce autophagy in glioma cells [3, 4]. Pathological studies have shown that the levels of Beclin 1 and LC3-2, biomarkers of autophagy, are lower in GBMs relative to lower-grade astrocytomas, as well as normal brain tissue [5, 6]. Furthermore, high Beclin 1 and LC3 levels were associated with improved survival in GBM patients [7, 8]. However, the specific role of autophagy in promoting the survival or death of brain tumors in various therapeutic settings remains largely unclear.

Melatonin is synthesized primarily in the pineal gland and may act as an anticancer agent [9, 10]. Melatonin inhibited the proliferation and enhanced the differentiation of tumor cell cultures [11–16]. Furthermore, it has been shown that melatonin decreases tumor growth in vivo [17, 18]. The antioxidant effects of melatonin have been considered to be the major mechanism underlying the antitumor properties of melatonin [14, 15]. However, it was recently proposed that melatonin exerts anticancer effects by activating the intrinsic and/or extrinsic apoptotic pathway in cancer cells [19].

Apoptosis-inducing therapies have gained interest as promising experimental treatment strategies for GBM. A previous report described an increase in glioma patient survival caused by a radio-neuroendocrine strategy using radiotherapy plus melatonin compared with radiotherapy alone [20]. Although several studies have shown that melatonin induces autophagy in colon cancer and hepatoma cells, there has been no direct evidence of whether melatonin is capable of inducing autophagy in human glioma cells. In the present study, we show that melatonin induced a completed autophagic flux in glioblastoma cells. Moreover, melatonin alone could not induce apoptosis, whereas disruption of autophagy triggered melatonin-induced apoptosis in glioblastoma cells.

## Results

**Fig. 1:**
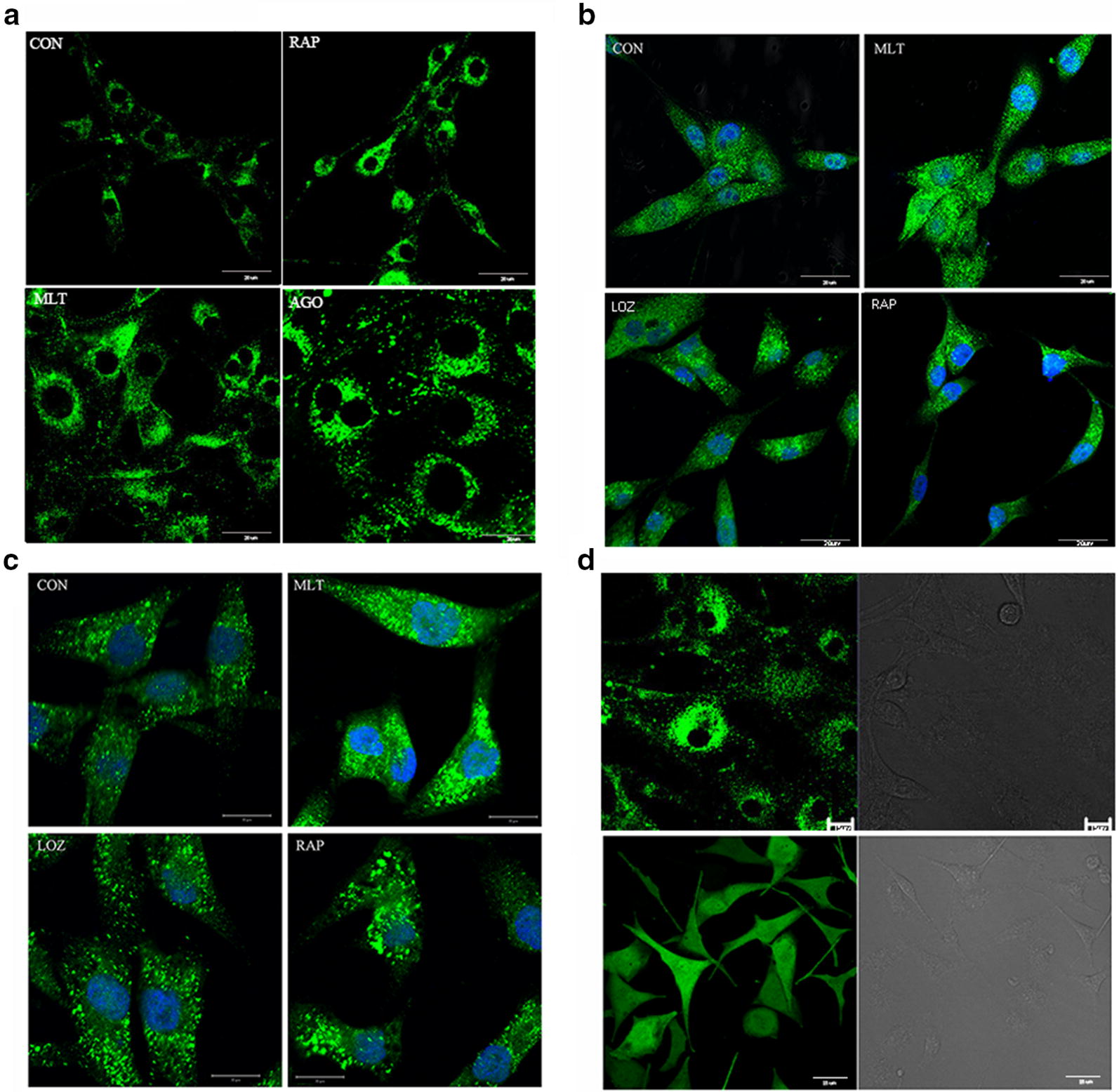
Melatonin induced LC3 aggregation and conversion. **a** GFP-LC3 aggregation and conversion in U87MG cells stably expressing an exogenous fusion protein, green fluorescent protein (GFP) and microtubule-associated light chain 3 (LC3) (U87-GFP-LC3), after treatment with DMSO (control), rapamycin (RAP 200 nM), melatonin (MEL 1 mM) or agomelatine (AGO 2 μM) for 24 h (×40); **b** U87MG cells and **c** A172 cells treated with DMSO (control), rapamycin (RAP 200 nM), melatonin (MEL 1 mM), agomelatine (AGO 2 μM) and luzindole (LUZ 5 μM/L) for 24 h were fixed and analyzed for endogenous LC3 dots by immunofluorescence using anti-LC3 antibodies (green), DAPI (blue) staining shows the nuclei (×40). **d** Stable U87-GFP-LC3 cell lines and GFP in vector control. U87MG cells stably expressing an exogenous fusion protein, green fluorescent protein (GFP) and microtubule-associated light chain 3 (LC3) (U87-GFP-LC3) (×40)

The levels of the microtubule-associated protein light chain 3 (LC3), an autophagosome protein, were used as an indicator of autophagy. To assess the autophagy-inducing ability of melatonin, we first established a U87MG cell line that stably expressed an exogenous fusion protein, green fluorescent protein (GFP), and microtubule-associated light chain 3 (LC3) (U87-GFP-LC3 cells) (Fig. 1d). In these cells, the GFP-LC3 was distributed evenly in the cytosol (Fig. 1a). Rapamycin, a common-used inducer of autophagy, caused extensive LC3 puncta (Fig. 1a). After incubating the cells with 1 mM melatonin for 24 h, there was a significant increase in the formation of LC3 dots (Fig. 1a). Treatment with agomelatine, an agonist of melatonin, also increased the punctate pattern of LC3 in the U87-GFP-LC3 cells (Fig. 1a). The effects of melatonin on the endogenous LC3 dot formation were further confirmed in both U87MG (Fig. 1b) and A172 cells (Fig. 1c). Interestingly, endogenous LC3 dot formation was significantly attenuated by treatment with luzindole, a nonspecific melatonin receptor antagonist in glioblastoma cells (Fig. 1b, c).

**Fig. 2:**
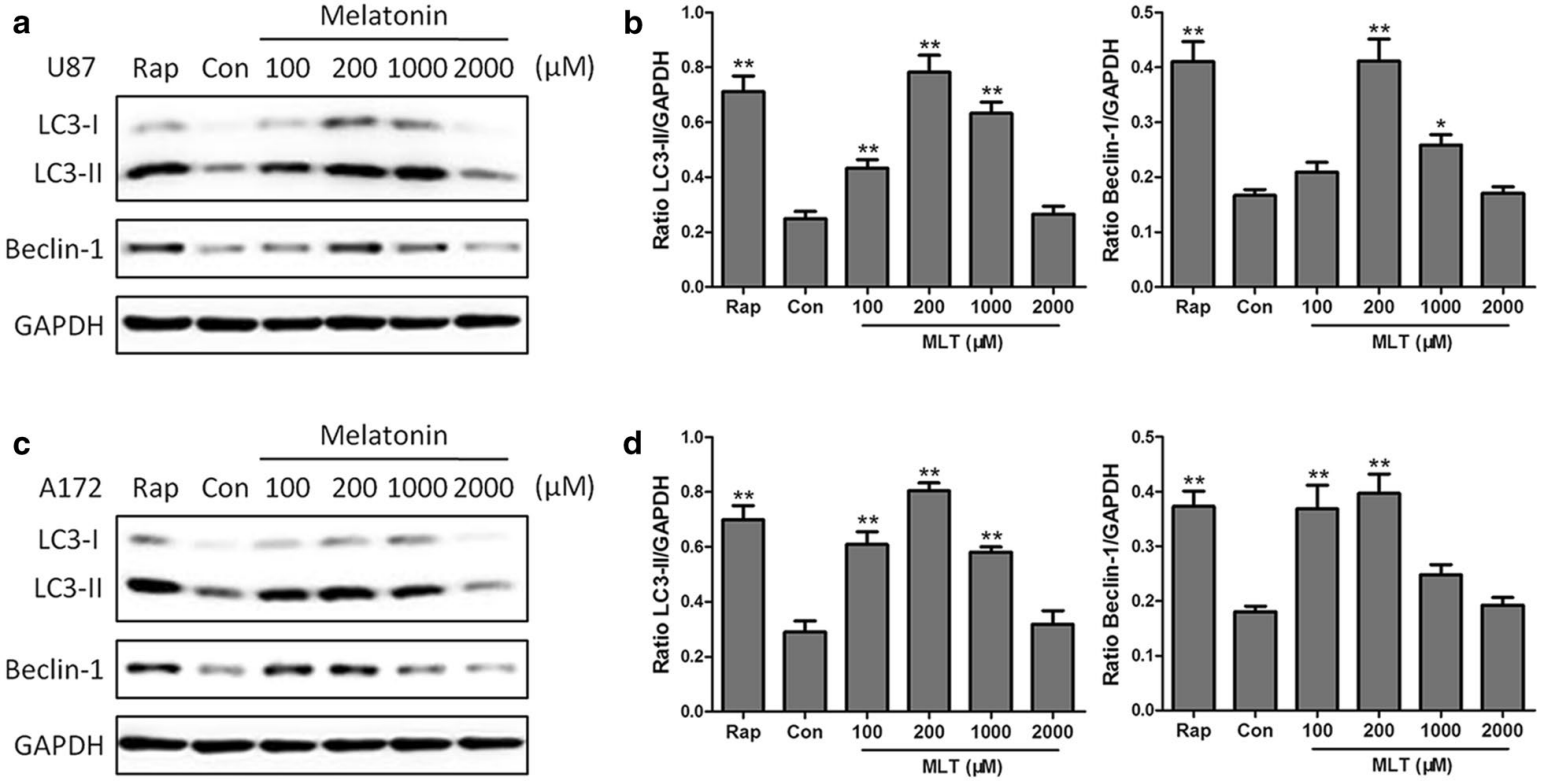
The concentration-dependent effects of melatonin on the LC3-II and Beclin-1 expression in U87MG (**a**, **b**) and A172 (**c**, **d**) cells. The cells were treated with 100, 200, 1000 or 2000 μM of melatonin for 24 h. PBS was used as a negative control, and rapamycin (Rap 200 nM) served as a positive control. The levels of LC3-II and Beclin-1 expression were determined by a Western blot analysis. The protein bands for each regimen were quantified by densitometry, and their differences are presented in the graph. The values are represented as the mean ± S.E.M. for three separate determinations. Statistical significance * at P < 0.05 or ** at P < 0.01 compared with untreated controls

Next, we tested the concentration- and time course-dependent effects of melatonin on the endogenous conversion of LC3-I to LC3-II and the expression of Beclin-1, based on Western blotting. First, we investigated the concentration-dependent effects of melatonin on the induction of autophagy in U87MG and A172 cells. U87MG cells or A172 cells were treated with 0, 0.1, 0.2, 1, and 2 mM of melatonin for 24 h. Upon treatment with 0.1 mM melatonin, the expression of LC3-II began to increase in both the U87 MG and A172 cells, and the maximum effect was achieved at 0.2 mM of melatonin (Fig. 2a–d). Beclin 1 is a key initiator of autophagy and is required for autophagosome formation during autophagy. A concentration-dependent effect of melatonin on Beclin 1 expression was also observed in U87MG and A172 cells. The results showed that Beclin 1 was significantly upregulated by melatonin at concentrations of 0.2 and 1 mM in U87MG cells and 0.1 mM to 0.2 mM in A172 cells (Fig. 2a–d). These results showed that melatonin significantly increased the expression levels of the LC3-II and Beclin-1 proteins in a concentration-dependent manner.

**Fig. 3:**
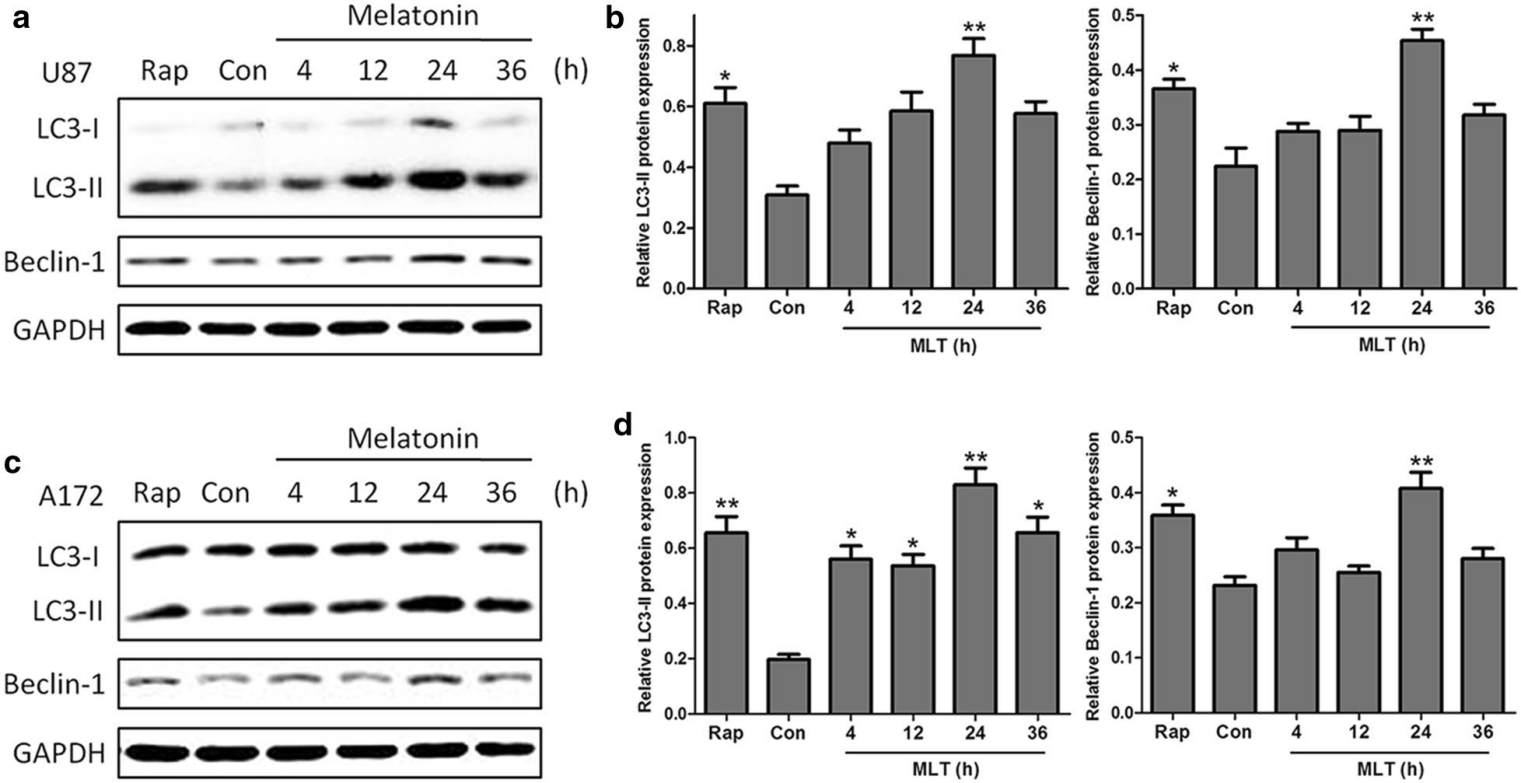
The time-dependent effects of melatonin on the LC3-II and Beclin 1 expression in U87MG (**a**, **b**) and A172 (**c**, **d**) cells. The cells were treated with 1 mM melatonin for 4, 12, 24 or 36 h. PBS was used as a negative control, and rapamycin (Rap 200 nM) served as a positive control. The levels of LC3-II and Beclin-1 expression were determined by a Western blot analysis. The protein bands for each regimen were quantified by densitometry, and their differences are presented in the graph. The values represent the mean ± S.E.M. for three separate determinations. Statistical significance * at P < 0.05 or ** at P < 0.01 compared with untreated controls

The effects of melatonin on the LC3-II and Beclin 1 expression were also time-dependent. Upon treatment with 1 mM melatonin for 4, 12, 24, or 36 h, the expression levels of both LC3-II and Beclin I were increased at 24 h post-treatment in both cells lines (Fig. 3a, b). Of note, melatonin showed an early effect on the LC3-II expression in A172 cells, with significant increases at 4, 12, and 24 h post-treatment (Fig. 3c, d).

**Fig. 4:**
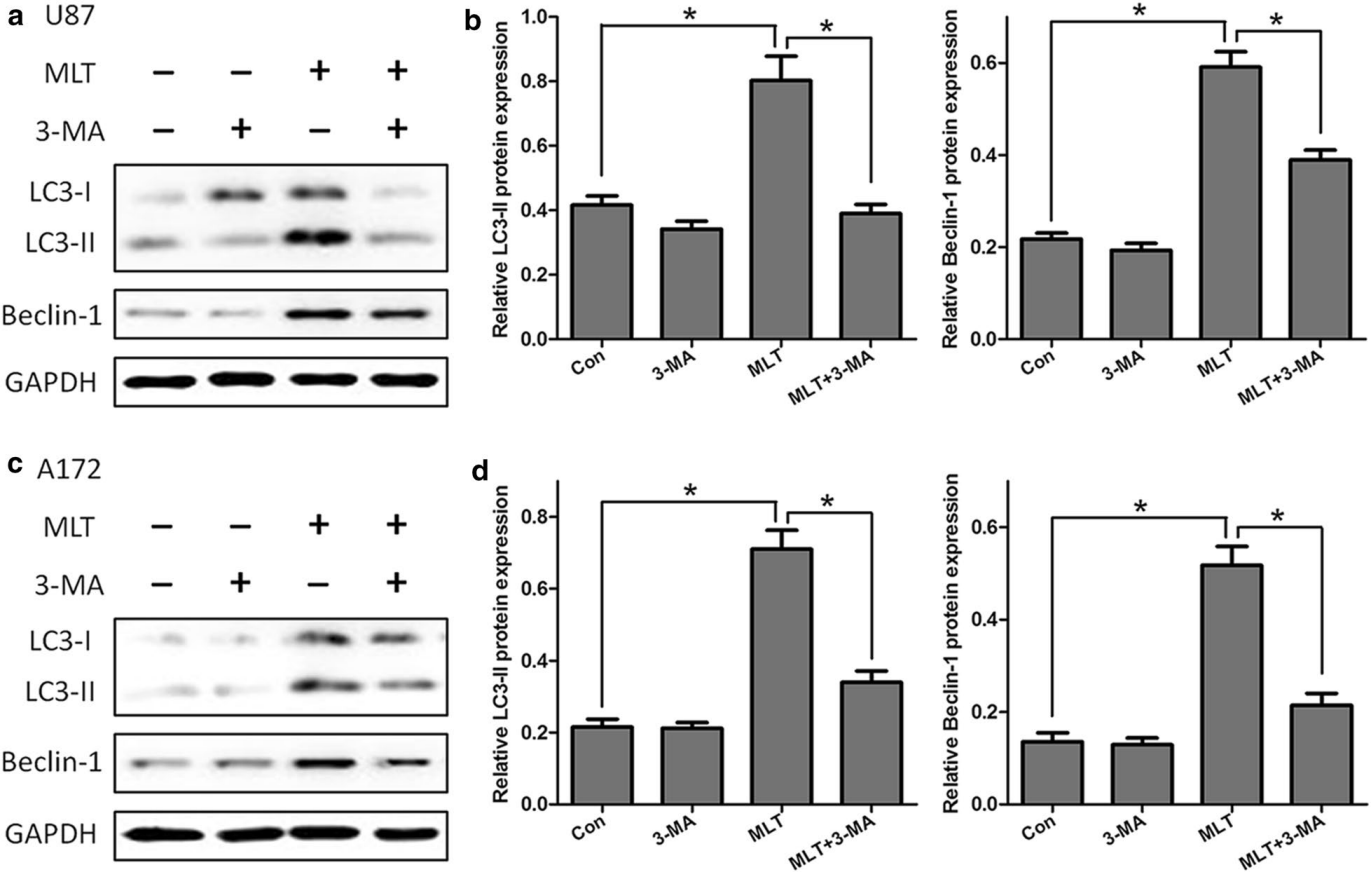
Melatonin-induced autophagy was abolished by treatment with an autophagy inhibitor, 3-MA. The results of the Western blotting of LC3 and Beclin-1 in the U87MG (**a**, **b**) and A172 (**c**, **d**) cells treated with PBS (control) or melatonin (MLT 1 mM) for 24 h in the presence and absence of 3-MA (10 mM). Statistical significance * at P < 0.05 compared with untreated controls or the corresponding group

**Fig. 5:**
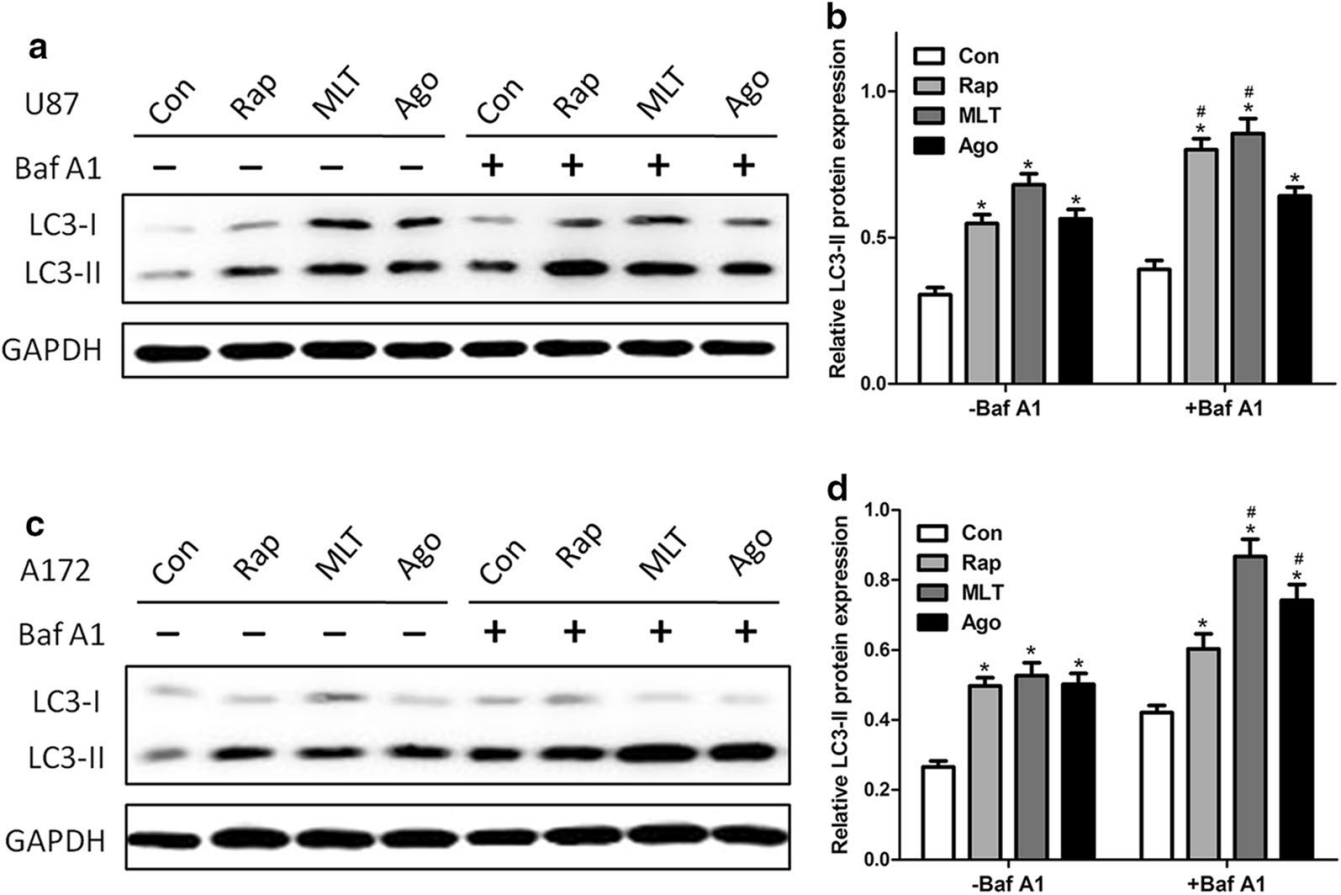
Melatonin induced autophagy by enhancing autophagosome formation. U87MG (A-B) and A172 (C-D) cells were treated with PBS (control), rapamycin (Rap 200 nM), melatonin (MLT 1 mM), or agomelatine (Ago 2 μg/ml) for 24 h in the presence or absence of 400 nM bafilomycin A1 (added 4 h before cell harvesting). The endogenous LC3-II levels were detected by Western blotting with anti-LC3 antibodies and were quantified by a densitometric analysis relative to GAPDH (**p < 0.01, compared to the control for each group, n = 3)

Because melatonin enhanced the formation of autophagosomes, we tested whether melatonin-induced autophagy could be inhibited by 3-methyladenine (3-MA), a widely used inhibitor of autophagy. As shown in Fig. 4 following pretreatment with 3-MA, the melatonin-induced LC3-II and Beclin I expression were significantly attenuated in both the U87 MG and A172 cells (Fig. 4). To further determine whether melatonin increased the autophagosome formation or decreased the autophagic degradation, bafilomycin A1 (Baf-A1), which inhibits the vacuolar H+ ATPase and prevents fusion between the autophagosome and lysosome, was used to measure the dynamic process of autophagy. We then tested the effects of melatonin in the presence and absence of Baf-A1, and the results are shown in Fig. 5. Melatonin and agomelatine significantly increased the LC3-II levels in U87MG and A172 cells in the presence of bafilomycin A1, strongly suggesting that melatonin enhanced the formation of autophagosomes (Fig. 5a–d). These results also suggested that the autophagic flux induced by melatonin was complete. In addition, extensive co-localizations were observed between the U87-GFP-LC3 dots and Lyso-Tracker Red, a selective dye that targets the lysosome, which strongly suggested that these U87-GFP-LC3 dots were probably from the fusion of autolysosomes with endo/lysosomes (Fig. 5e). Moreover, mitochondria (stained by MitoTracker) also co-localized with GFP-LC3 (Fig. 5f), indicating that the autophagosome/autolysosome contained mitochondria in the U87MG cells.

**Fig. 6:**
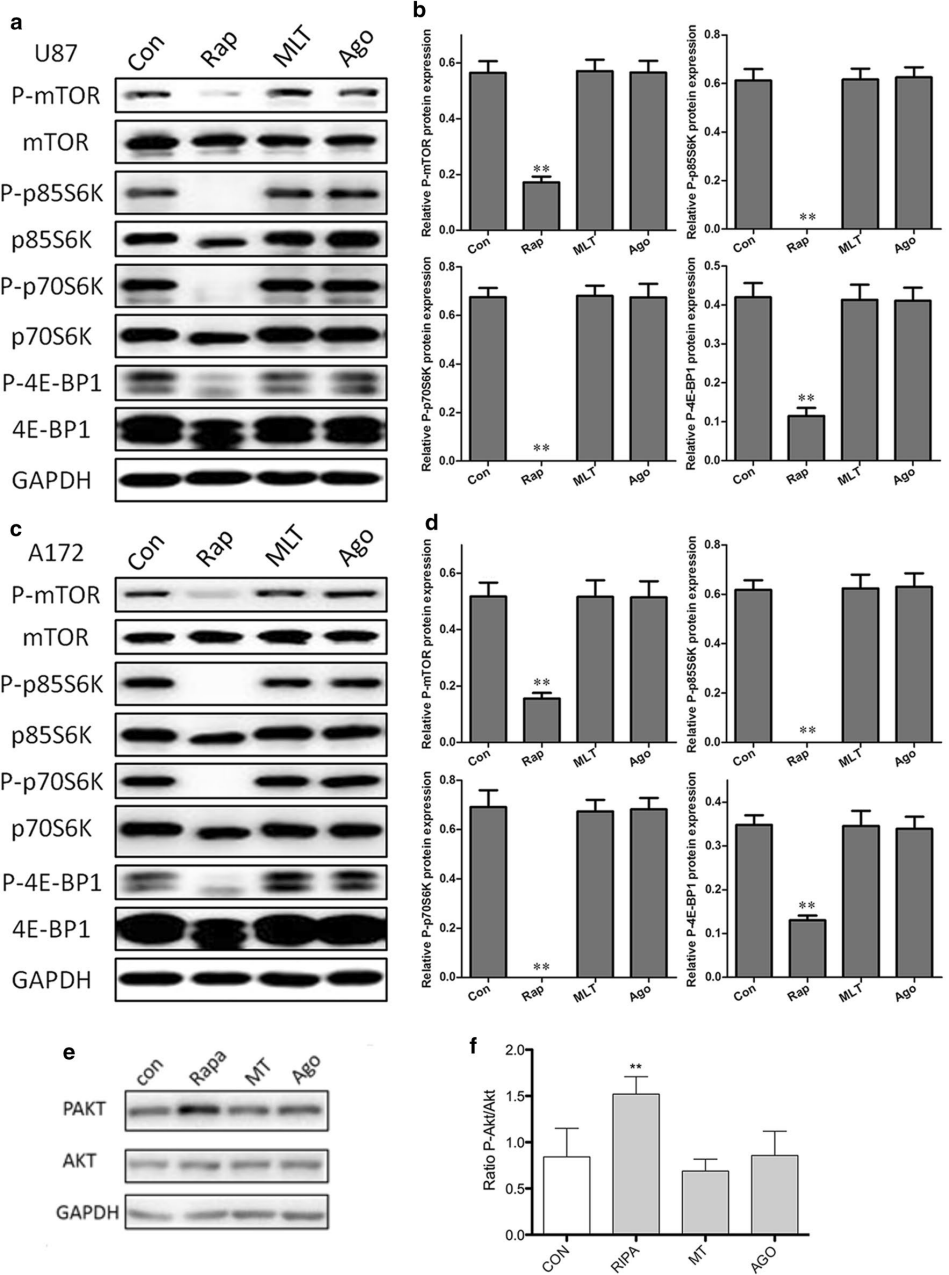
Melatonin-induced autophagy is not dependent on the mTOR signaling pathway. The U87MG (**a**, **b**) and A172 (**c**, **d**) cells treated with PBS (control), rapamycin (200 nM, a positive control), melatonin (1 mM) and agomelatine (2 μg/ml) for 24 h were analyzed for mTOR activity by Western blotting to determine the levels of total and phospho-mTOR, total and phospho- p85S6k, p70S6K and 4E-BP1

To gain insight into the signaling pathway underlying the induction of autophagy by melatonin, we investigated whether the PI3K/Akt/mTOR pathway was involved in this process. mTOR is a well-known negative regulator of autophagy. We also assessed the phosphorylation status of two well-characterized substrates of mTOR, ribosomal S6 protein kinase (p70S6K/p85S6K) and eukaryotic initiation factor 4E binding protein 1 (4E-BP1), to assess the mTOR activity. As shown in Fig. 6, unlike rapamycin (a specific mTOR inhibitor), melatonin did not reduce the phosphorylation level of mTOR or its substrates, ribosomal S6 protein kinase (p70S6K/p85S6K) and eukaryotic initiation factor 4E-binding protein1 (4E-BP1) in either of the glioblastoma cell lines. We further analyzed Akt signaling, which is upstream of mTOR. Consistent with the lack of changes in mTOR activity, there were no changes in the phosphorylation status of Akt following the treatment with melatonin or agomelatine. These results indicated that at these concentrations, the melatonin-induced autophagy occurs in an mTOR-independent manner in U87MG and A172 cells (Fig. 6).

**Fig. 7:**
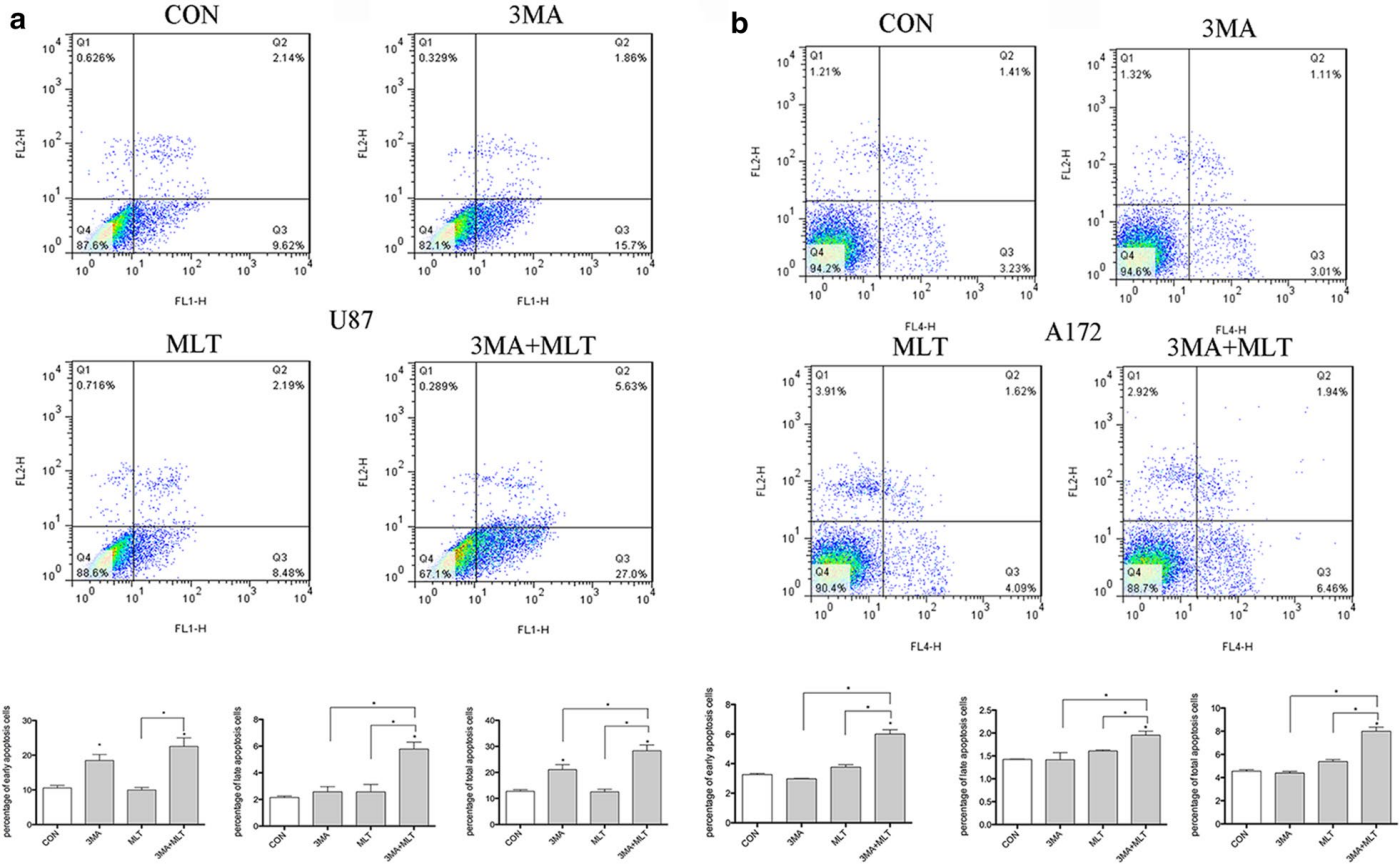
Inhibition of autophagy triggers melatonin-induced apoptosis in glioblastoma cells. **a** U87MG cells. **b** A172 cells. The cells were treated with DMSO as a negative control, 3-MA (10 mM), or melatonin (1 mM), or were co-treated with 3-MA and melatonin for 24 h. The 3-MA was added to the medium 1 h prior to the administration of melatonin. Cell death was determined by flow cytometry following Annexin V/PI staining. The percentages of cells in early and late apoptosis and the total number of apoptotic cells are presented as the mean ± S.D. from three independent experiments. Statistical significance *P < 0.01 vs controls or the corresponding group

**Fig. 8:**
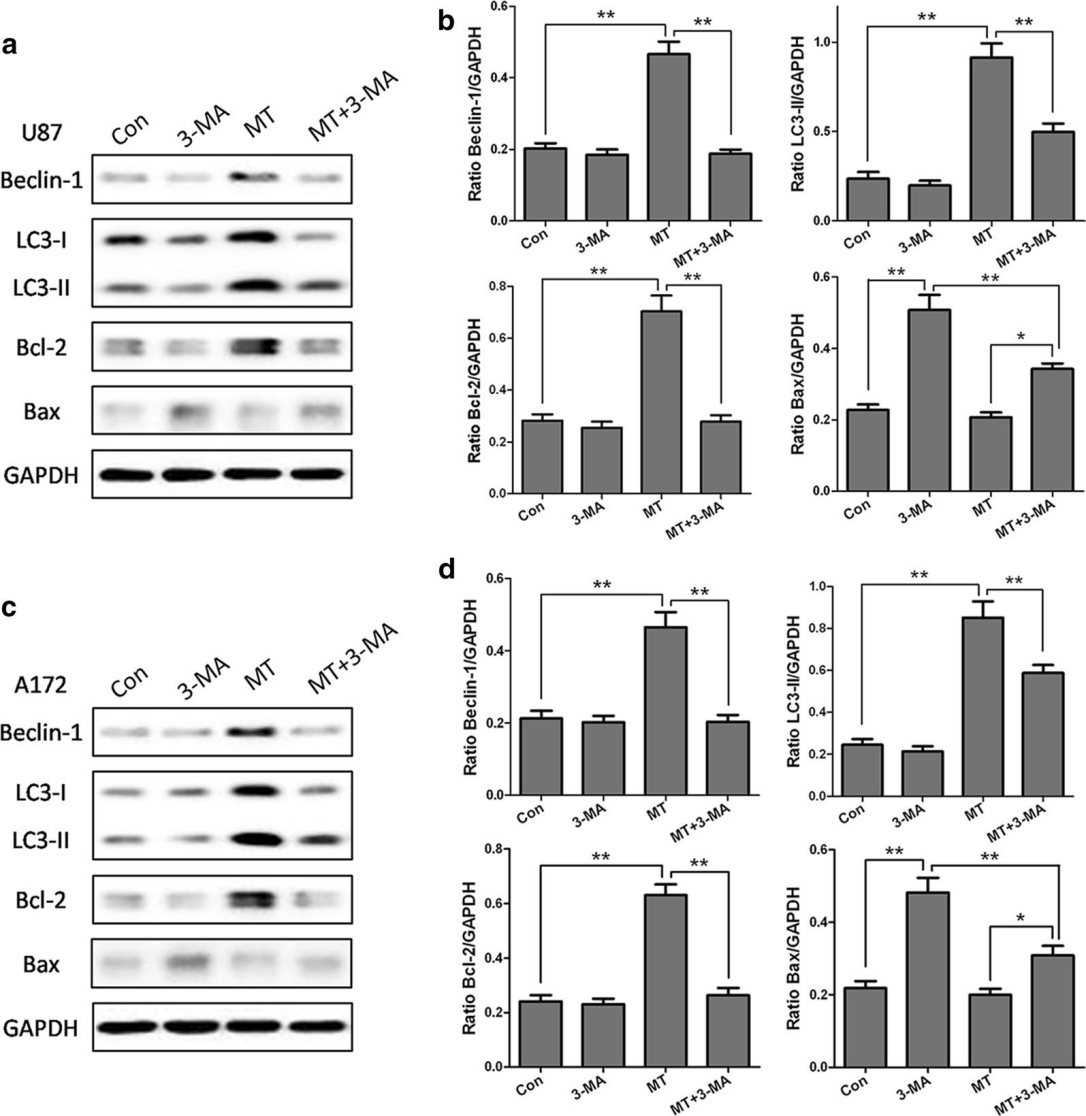
Inhibition of autophagy by 3-MA triggers melatonin-induced activation of apoptotic proteins in glioblastoma cells. The protein expression levels of Beclin 1, LC-3, Bcl-2 and Bax were assessed in U87MG (**a**, **b**) and A172 (**c**, **d**) cells by Western blotting. The cells were treated with DMSO as a negative control, 3-MA (10 mM), or melatonin (1 mM) or were co-treated with 3-MA and melatonin for 24 h. The 3-MA was added to the cell culture medium 1 h prior to the administration of melatonin. The protein bands for each treatment regimen were quantified by densitometry, and their differences are presented in the graph. The values represent the mean ± S.E.M. for three separate determinations. Statistical significance *P < 0.05 and **P < 0.01 compared with the corresponding groups

To investigate whether there is crosstalk between the melatonin-induced autophagy and apoptosis in glioblastoma cells, we evaluated the consequences of disrupting autophagy by treating cells with 3-MA (an inhibitor of autophagy). As shown in Fig. 7, melatonin alone did not induce any changes in the apoptotic population in either U87MG or A172 cells (Fig. 7a, b). To inhibit autophagy, cells were pretreated with 3-MA (10 mM) prior to the administration of melatonin (1 mM). Compared with treatment with melatonin alone, co-treatment with melatonin and 3-MA significantly enhanced the apoptotic cell population in both U87MG and A172 glioblastoma cells. A Western blot analysis confirmed that melatonin treatment alone prominently upregulated the level of Beclin 1, a key initiator of autophagy, and LC3 II, a central component of autophagosome formation, along with a two-fold increase in the expression of Bcl-2, an anti-apoptotic protein, in both glioblastoma cell lines (Fig. 8). Consistent with the effects on apoptosis, treatment with melatonin alone had no effect on the expression of Bax, an apoptotic protein. While 3-MA treatment alone had no effect on the expression of Beclin 1 and LC-3, pretreatment with 3-MA prominently abolished the melatonin-induced enhancement of Beclin 1 and LC-3 (Fig. 8). Further studies showed that co-treatment with melatonin and 3-MA prominently decreased the expression of the anti-apoptotic protein, bcl-2, whereas the expression of an apoptosis-promoting protein, Bax, was significantly increased, which collectively indicated that the disruption of autophagy triggers the melatonin-induced apoptosis in glioblastoma cells (Fig. 8).

## Discussion

In the present study, we reported that melatonin or its agonist, agomelatine, induced the complete flux of autophagy in glioblastoma cells, which was significantly attenuated by treatment with luzindole, a melatonin receptor antagonist. Furthermore, we showed that melatonin treatment alone did not induce any apoptotic changes in the glioblastoma cells, whereas co-treatment with an autophagy inhibitor, 3-MA, in combination with melatonin, significantly enhanced the apoptotic cell population in two glioblastoma cell lines.

The role of autophagy in cancers is controversial because it appears to be able to act as either a tumor suppressor or promoter depending on the tumor type and stage. In the context of brain tumors, autophagy may play a role as a tumor suppressor role, as mutations of tumor suppressors known to positively regulate autophagy have frequently been reported [2]. Several studies have shown that melatonin induces autophagy in colon cancer [21–23] and hepatoma cells [24]. In the present study, we reported that melatonin or its agonist, agomelatine, induces autophagy in glioblastoma cells. Furthermore, by suppressing autophagy at an early stage with 3-MA and at a late-stage with bafilomycin A1, we found that the melatonin-induced autophagy was activated early and the autophagic flux was complete.

In glioma cells autophagy has been proposed to be an adaptive response to treatment with both radiation and temozolomide, suggesting that inhibition of autophagy could have therapeutic benefits [3, 25–27]. However, phase II studies with temsirolimus, an analog of the allosteric mTOR inhibitor, rapamycin, did not provide any survival benefit in patients with recurrent GBM [28, 29]. Moreover, a recent phase I/II trial of hydroxychloroquine in conjunction with radiation therapy and temozolomide in patients with newly diagnosed glioblastoma multiform showed that there was no significant improvement in the overall survival [30]. On the other hand, low levels of Beclin 1 and the LC3-II protein have been found in higher-grade astrocytomas, suggesting that a decrease in autophagic activity may drive the progression of astrocytic tumors [6]. In this regard, the induction of autophagy by melatonin or agomelatine may represent a potential therapeutic strategy for glioma. Indeed, it has recently been shown that melatonin treatment promotes cell differentiation and induces cell death with ultrastructural features of autophagy in glioblastoma-initiating cells (GIC) [31]. The induction of autophagy by rapamycin also promoted the differentiation of GIC isolated from human GBMs, and this was followed by a reduction in cell proliferation [32]. In addition, autophagic cell death was also observed in the GICs when they were treated with cell-penetrating D-isomer peptides [33]. It is worthwhile to mention that agomelatine, the agonist of melatonin, has recently been proposed as a treatment adjunct in glioblastoma because it showed advantages over the natural ligand, such as a longer half-life, better oral absorption, and higher affinity for melatonin receptors [34]. Thus, the induction of autophagy by agents such as melatonin, rather than inhibition of autophagy, may represent a promising strategy that may lead to new treatment for glioma.

With respect to the signaling pathways involved in the melatonin- and agomelatine-induced autophagy, we found that these effects were independent of the PI3K/AKT/mTOR/p70S6K signaling pathway in glioblastoma cells. The PI3K/Akt/mTOR pathway is an important negative signaling pathway involved in autophagy. Of note, inhibition of the PI3K/Akt/mTOR pathway induces autophagy in human malignant glioma cells [35]. It was also demonstrated that inhibition of Akt, but not ERK1/2, is involved in the inhibition of glioma cell proliferation following treatment with melatonin [36]. Melatonin paradoxically induces either inhibition or activation of the PI3K/Akt/mTOR pathway. Melatonin-induced autophagy via the PI3K/Akt/mTOR pathway has been observed in a number of different cancer cells, such as hepatoma cells [24, 37]. In contrast, Kongsuphol et al. showed that treatment with melatonin enhanced the mTOR activity in the SK-N-SH dopaminergic cell line [38]. Zheng et al. also showed that melatonin inhibits autophagy by activating the PI3K/Akt pro-survival pathway in a rat model of transient focal cerebral ischemia [39]. Xiao et al. demonstrate that melatonin activates transcriptional coactivator peroxisome proliferator-activated receptor gamma coactivator 1A (PGC1A) and uncoupling protein 1 (UCP1)-dependent lipid autophagy thus suppressing tumor progression [40]. In the present study, we did not find any changes in the PI3K/Akt/mTOR pathway in our experiments. The differences in the findings may be due to the different cancer types and/or the concentrations or doses used in the experimental model. On the other hand, it may also indicate that other signal pathway(s) may be involved in the melatonin-induced autophagy in glioblastoma cells.

It has recently been explored whether it is possible to modulate autophagy to produce a synergistic effect on tumor cell death by inducing apoptosis, providing a potential combination therapy for glioma [2, 41]. Although several studies have shown that melatonin induces apoptosis in other types of cancer cells, such as prostate and gastric cancer cells, there is no direct evidence regarding whether melatonin can induce apoptosis in glioma cells, such as U87MG or A172 cells. An early report showed the induction of apoptosis by high concentrations of melatonin after 72 h of treatment in a human neuroblastoma cell line, SK-N-MC [9]. In the present study, melatonin alone did not induce apoptosis in the glioblastoma cells. Consistent with our findings, there was also no apparent induction of apoptosis after melatonin treatment in GICs [31]. Remarkably, in the current study, we found that the inhibition of autophagy by 3-MA triggered melatonin-induced cell apoptosis, along with a significant upregulation of the levels of a pro-apoptotic protein, Bax, in the glioblastoma cells. Bcl-2 and Bax provide a molecular link between autophagy and apoptosis. In the present study, we found that melatonin alone induced an increase in bcl-2 expression to a level. When autophagy was inhibited by 3-MA pretreatment, the melatonin induced increase in bcl-2 was completed blocked. In turn, melatonin induced a significant upregulation of the levels of the pro-apoptotic protein Bax.

## Conclusion

The present study showed that melatonin or its agonist, agomelatine, can activate cell autophagy, which may be important for glioma cell differentiation. The inhibition of autophagy triggered melatonin-induced apoptosis in glioblastoma cells. Collectively, these results provide information regarding the mechanism by which melatonin may act as a common upstream signal between apoptosis and autophagy, which may lead to the development of new therapeutic strategies for glioma.

## Methods

### Antibodies and reagents

Anti-microtubule associated protein 1 light chain 3 (LC3) (NB100-2220) and anti-Beclin 1 (ab55878) antibodies were purchased from Novus Biological (LLC, USA). Anti-Bcl-2-associated X protein (Bax) and anti-Bcl-2 antibodies were purchased from Abcam (Cambridge, MA, USA). Antibodies recognizing phospho-mTOR (Ser2448), mTOR, phospho-4E-BP1 (Thr37/46), 4E-BP1, phospho-p85S6K (Thr412), p85S6K, phospho-p70S6K (Thr389), and p70S6K were purchased from Cell Signaling Technology (Beverly, MA, USA). Lipofectamine 2000 (11668-019), LysoTracker Red (L7528) and MitoTracker (M7512) were purchased from Invitrogen. The β-actin (KC-5A08) and GAPDH (KC-5G5) antibodies were from Kangcheng (Shanghai, China). Melatonin and all other reagents were purchased from Sigma Chemical Co. (St. Louis, Missouri) unless otherwise indicated.

### Cell culture

The U87 MG and A172 cell lines were obtained from the Shanghai cell bank and cultured with Dulbecco’s modified Eagle’s medium (DMEM/F12) (Sigma) supplemented with 10% fetal bovine serum (FBS) (GIBCO, Grand Island, New York), 10 units/ml penicillin, and 10 µg/ml streptomycin at 37 °C in a humidified 5% CO_2_ atmosphere. U87 MG-LC3 stable cell lines were generated from cells that had been transfected with the EGFP-LC3 plasmid using Lipofectamine 2000 (Invitrogen) according to the manufacturer’s recommendations. Stably transfected EGFP-LC3 cells were selected in DMEM/F12 containing 10% FBS and 600 µg/ml G418 (Invitrogen). The LC3 expression level was determined by confocal microscopy.

### Immunofluorescence staining

Immunofluorescence staining was performed as described previously [42]. U87MG and A172 cells were fixed with 4% paraformaldehyde in TBS (tris buffered saline: 0.05 m Tris, 0.9% NaCl, pH 7.6) for 15 min at room temperature, rinsed in TBS (tris-buffered saline: 0.05 m Tris, 0.9% NaCl, pH 7.6) thee times for 10 min each, and were treated with 0.3% hydrogen peroxide in TBS for 30 min to quench endogenous peroxidase activity. We used 1% triton-1000 for permeabilization. The anti-LC3 (1:300) antibodies were added, and the samples were incubated overnight at 4 °C.

### Dye loading

After being treated with melatonin, agomelatine or rapamycin for 24 h, the U87 MG cells were treated with 10 μM monodansylcadaverine, 20 nM Mito Tracker Red and 75 nM LysoTracker Red for 30 min, respectively. All imaging was performed on a Zeiss LMS710 confocal microscope, and the data were processed as described previously [42].

### Western blot analysis

The U87MG and A172 cells were homogenized in radioimmunoprecipitation assay (RIPA) buffer [50 mM Tris–HCl (pH 7.4), 0.1% SDS, 1% Triton X-100, 0.25% sodium deoxycholate, 150 mM NaCl, 1 mM EDTA, 1 mM EGTA, and 1 mM Na3VO4] containing 1 mM phenylmethylsulfonyl fluoride (Amresco, Solon, Ohio, USA) and protease inhibitor cocktail (Roche, Indianapolis, IN, USA). The protein samples were separated on a 12% SDS polyacrylamide gel and detected with antibodies against LC3, Beclin-1, Bax, Bcl-2, phospho-mTOR/mTOR, phospho-4E-BP1/4E-BP1, phospho-p85S6K/p85S6K, or phospho- p70S6K/p70S6K. β-actin (KC-5A08, Kangcheng, China) or GAPDH (KC-5G5, Kangcheng, China) were used as a loading control, and then the blots were probed with appropriate HRP-conjugated secondary antibodies. The protein bands were detected by enhanced chemiluminescence (ECL) (Amersham Bioscience, UK) and quantified using the Image J software program (NIH).

### Flow cytometric analysis of apoptosis

The U87MG or A172 cells were seeded in 6-well plates at a density of 1× 10^6^ cells/well. The cells were treated for 24 h with 1 mM melatonin or 10 mM 3-MA. Afterwards, the cells were washed in PBS and stained with FITC-conjugated Annexin V and propidium iodide (BD Biosciences) in binding buffer. The cells were incubated at room temperature for 15 min in the dark and analyzed by a Beckman Coulter FC500 flow cytometer (Beckton Dickinson, Franklin Lakes, NJ, USA) using the WinMDI2.9 software program.

### Statistical analysis

The statistical analysis was performed with the SPSS software program (SPSS Software, Chicago, IL, USA). The differences between the groups were evaluated by a two-tailed Student’s *t* test or a one-way ANOVA, as appropriate. Throughout this study, the values are expressed as the mean ± SEM. Values of P < 0.05 were considered to be significant.

## Data Availability

The datasets used and/or analysed during the current study are available from the corresponding author on reasonable request.

## Abbreviations

GBMs: glioblastomas
RT: radiation therapy
TMZ: temozolomide
LC3: anti-microtubule associated protein 1 light chain 3
BAX: anti-Bcl-2-associated X protein
mTOR: mammalian target of rapamycin
DMEM: Dulbecco’s modified Eagle’s medium
FBS: fetal bovine serum
TBS: tris buffered saline
RIPA: radioimmunoprecipitation assay
ANOVA: analysis of variance
GFP: green fluorescent protein
GIC: glioblastoma-initiating cells
